# Challenges of Obtaining Informed Consent in Emergency Ward: A Qualitative Study in One Iranian Hospital

**DOI:** 10.2174/1874434601711010263

**Published:** 2017-12-29

**Authors:** Nayyereh Davoudi, Nahid Dehghan Nayeri, Mohammad Saeed Zokaei, Nematallah Fazeli

**Affiliations:** 1 Department of Medical Surgical Nursing, School of Nursing and Midwifery, Tehran University of Medical Sciences, Tehran, Iran; 2 Nursing and Midwifery Care Research Center, School of Nursing and Midwifery, Tehran University of Medical Sciences, Tehran, Iran; 3 Faculty of Social Sciences, Allameh Tabataba'i University, Tehran, Iran; 4 Institute for Humanities and Cultural Studies, Tehran, Iran

**Keywords:** Informed consent, Emergency ward, Qualitative content analysis, Medical paternalism, Healthcare providers

## Abstract

**Background and Objective:**

Regarding the fact that emergency ward has unique characteristics, whose uniqueness affects informed consent processes by creating specific challenges. Hence, it seems necessary to identify the process and challenges of informed consent in the emergency ward through a qualitative study to understand actual patients’ and health care providers’ experiences, beliefs, values, and feelings about the informed consent in the emergency ward. Through such studies, new insight can be gained on the process of informed consent and its challenges with the hope that the resulting knowledge will enable the promotion of ethical, legal as well as effective health services to the patients in the emergency ward.

**Method:**

In this qualitative study, research field was one of the emergency wards of educational and public hospitals in Iran. Field work and participant observation were carried out for 515 hours from June 2014 to March 2016. Also, conversations and semi-structured interviews based on the observations were conducted. The participants of the study were nurses and physicians working in the emergency ward, as well as patients and their attendants who were involved in the process of obtaining informed consent.

**Results:**

Three main categories were extracted from the data: a sense of frustration; reverse protection; and culture of paternalism in consent process.

**Conclusion:**

Findings of this study can be utilized in correcting the structures and processes of obtaining informed consent together with promotion of patients' ethical and legal care in emergency ward. In this way, the approaches in consent process will be changed from paternalistic approach to patient-centered care which concomitantly protects patient’s autonomy.

## 1 INTRODUCTION

### 1.1 Background

The concept of “consent” is a little different in various cultures and countries, For example according to the varieties of differing regulations and contexts. For example, according to the American laws, it emphasizes the patient’s autonomy. The model’s building blocks consists of competence, disclosure, understanding, voluntariness and consent [1]. Henceforth, the emphasis of the consent lies on the duty of the physician to disclose information to the patient. The patient, in this model, is the lone person who knows his/her conditions and situation in the best way possible, with the physician as a person committed to and responsible for the provision of patient’s information needs in order to help the patient make the optimal decisions.

However, in the UK, it is the valid consent which is applied in those consent processes. It is defined in terms of its detailed reference to the patient and is regarded as valid when it has the following characteristics:

1. The patient consents voluntarily, without any sort of coercion
2. The patient has the capacity and competence to consent
3. The patient possesses the least information required to consent about the nature of the procedure he is going to experience [2].

On the other hand, informed consent in Iran is treated as basic and indispensable rights of the patient. It is regard as the basis and fundamentals of ethics in medicine [3]. Parsapour *et al* (2005), mentioned that informed consent is a qualified person's unconditional and free agreement on involvement in decision making. It originates from awareness in its nature, purpose and consequences of their decisions with the belief that it will lead to the most effective and beneficial treatment [4]. Therefore, it is an ethical and legal process with the aim of promoting patient's health and respecting patients' independence and autonomy [5]. It is discussed in depth in ethical coding of patient rights in Iran; accordingly, in one of the code of ethics, it is mentioned that the choice and decision making by patient should be deliberate, free and based on the comprehensive information received. As a result, diagnostic and treatment procedures and benefits besides their side effects, the nature of disease, prognosis coupled with any required information in the decision making process should be provided to the patient. The information, should be provided in the appropriate time and on the best condition of the patient free from stress or pain and personal features such as language, education as well as his capability to understand. If delay in treatment for the sake of providing the information causes injuries to the patient, in such situations, emergency treatment is provided, then the information is given in due time. Meanwhile it is emphasized in this code that enough time should be given to the patient so as to make a choice and decision. Accordingly, the physician is enquired to provide required information to the patient in an explicit and clear, accurate and understandable way [6]. Furthermore, Informed consent has been referred to as an important legal matter in Iran’s laws. As it is mentioned in the article 158 of Islamic Penal Act, every type of surgery or medical treatments which is done with the consent of the patient or his legal guardians, authorities or representatives; and based on scientific and technical standards and government regulations isn’t regarded as crime. It is also mentioned that informed consent is not required in emergency situations. As a result, in cases where there is no received informed consent in non-emergency situations and the patient experiences injuries, he has the right to ask for financial compensations from the physician based on Islamic Penal Act [7].

Therefore, it can be said that informed consent is a legal process meaning that the patient or his/her surrogate is informed of the benefits, risks or other alternatives of treatment, and thus by signing the form, provides a legal document [8]. Henceforth the necessity for signing the form before an operation is undeniable and the most legally evident and ethical right of the patient [9].

### 1.2 Importance and Study Objective

Researcher's experience about informed consent in Iranian hospitals, talks of the patients signing the forms unread with the healthcare teams not giving enough explanations about the upcoming procedures and their future aftermaths. On the other hand, review of the literature on the conducted studies in this realm indicates that majority of hospitals in Iran suffer big challenges in this area, as an example, in a descriptive study conducted by Taghaddosinejad *et al* (2008), in surgery wards of one of the Tehran's hospitals, showed that only half of the patients participating in the study had received required information prior to their surgery. Also, the degree of their awareness on the success rate of the surgery was 44.5%, probable side effects was 54.3% and their awareness of the other treatment alternatives was 61.4% [10]. Also, the results of the study done by Zafarghandi *et al* (2014), in surgery wards in one of Tehran hospitals indicated that the forms were understandable only to 19.5% of the patients. Also, 8.9% of the patients did not receive any information about their disease, 11.8% about the type of treatment and 47.3% about probable side effects of the surgery [11]. The results of another study done by Faghanipour and his colleagues on 300 patients undergoing surgery in seven educational hospitals also indicated that insufficient information was provided to patients about the nature of the disease, type of surgery, benefits and importance of surgery along with problems of not having the surgery [12].

Furthermore studies in other countries showed similar results. For example Özhan *et al* (2014), in their study concluded that at the pre-anesthetic visit, 54.8% of the 522 patient did not read the informed consent forms after signing [13]. Also in a study conducted by McGaughey (2004), in Australia, results showed that the little information was given to the patients on possible side effects and post-operative care of knee arthroscopy [14]. In other studies the consent forms as well as procedures were not understandable for the patients [15-17]. Also, researchers indicated that there are many factors that influence the process such as time, intelligence, educational degree, sex, and obtaining consent through interpreters [18-20].

The researcher's experience coupled with a review of the literature done in Iran and other countries showed that informed consent processes face serious challenges in various hospital wards. However, this process in emergency ward has not been extensively studied. It seems that the informed consent process in emergency wards is different from what happens in other wards, the reason being the characteristics of the emergency ward in which the number of patients is increasing continuously. This condition leads to frequent decision making in a short period of time [21]. In addition, the patients in the emergency ward often don’t have familiarity with the clinical environment, rarely have previous relationships with emergency health care providers, and may be acutely ill, weakened, or in much pain [22]. Also, they are at the beginning of receiving health and medical services [23], therefore, the decisions made in this phase can have dramatic consequences on the patients’ personal life [24]. Given that emergency ward has unique characteristics, it seems that this uniqueness affects informed consent processes creating specific challenges. Hence, it seems necessary to identify the process and challenges of informed consent in the emergency ward through a qualitative study to understand actual patients’ and health care providers’ experiences, beliefs, values, and feelings about the informed consent in the emergency ward. Through such studies, new insight can be gained on the process of informed consent and its challenges with the hope that the resulting knowledge will enable the promotion of ethical, legal as well as effective health services to the patients in the emergency ward. As a result, this study was done to answer to these questions:

How is the process of informed consent in the emergency ward?

What are the challenges of informed consent in the emergency ward?

What beliefs, values and norms have prepared the ground for the emergence of informed consent challenges?

## 2 METHODS

### 2.1 Study Design

The current study employed a qualitative study to access a deeper understanding of the informed consent processes in the emergency ward. According to Krippendorff (1980), as cited in Elo and Kyngäs, qualitative content analysis is a suitable method to derive replicable and valid inferences affected by a deep understanding of the context. It creates new knowledge and insight, a representation of facts and a practical guide to performance [25]. Kyngäs and Vanhanen (1999) believed that “The purpose of content analysis is to achieve a condensed and broad description of the phenomenon, and the outcomes of the analysis are concepts or categories describing the phenomenon” [26].

### 2.2 Research Field and Participants

In the present study, the research field was one of the emergency wards of educational and public hospitals in Iran. Given that the hospital is one of the main reference centers for emergencies and referred patients have various qualities from the economic, social, and cultural characteristics, it appears to be a suitable field for the present study. In this ward there were forty-four fixed beds for hospitalization which could be extended to up to thirty more beds in cases of necessity and more demand. On average 3618 patients were admitted to the emergency department monthly. Each work shift was run by 25 nurses, 3 medical interns, 6 emergency residents, and one emergency specialist and three secretaries. In the emergency ward of this study, the patients were classified in the triage based on the ESI (Emergency Severity Index) criteria. Then patients in level one (patients require immediate life - saving intervention), were immediately transferred from triage to a CPR (Cardiopulmonary Resuscitation) unit. It should be noted that according to article 158 of Islamic Penal Act, obtaining informed consent in emergency conditions is not required [7], as a result, the present study does not cover the process in the CPR unit.

The participants and informants of the study were purposely selected. They were nurses and physicians working in the emergency ward, patients and their attendants (most of them being patients' family members or relatives), and those who presented in the emergency ward and were involved in the process of obtaining informed consent.

It should be noted that some of the cultural characteristics and specific rules of the research field were considered as factors influencing the process of obtaining consent. For example, one of the features related to Iranian culture and the context of research field was the permanent presence of the patient's attendance on the patient's bedside, as it also affected the process of obtaining informed consent. In Iranian culture, there is a close family relationship between family members and most of the personal decisions are shared with families, especially in critical conditions when a member is ill. In such conditions, the immediate members of the family do their best to make the best decisions and take care of the affected family member. In hospitals, particularly in emergency wards, there is generally one family member accompanying patient and he accompanies him until the end of the treatment process, providing primary care for the patient. After discharge, family has also the responsibility for home care. Therefore, the patient attendant’s roles are highlighted in Iranian hospitals. Legally, according to the Iranian laws, in special conditions, informed consent can be obtained from his legal guardians, authorities or representatives [7]. As a result, by observing the mentioned conditions, obtaining informed consent from the patients' attendance for the purposes of medical procedures finds a legal format.

In addition to general laws, especial emphasis was given to the informed consent prior to specific invasive procedures in the emergency ward where the study was carried out. In one of the guidelines within the emergency ward, it is emphasized that informed consent is mandatory about invasive procedures. In such cases, implied consent does not suffice. In other words, implied consent was acceptable in routine cases such as taking medical history or vital signs. The following text refers to this guideline:

**Dear Physicians and Nurses**

**Subject**: Informed consent from the patients

Informed consent is carried out by physicians and nurses and each has duties as follows:

**Physician**: The physician conducting therapeutic or diagnostic non-emergency procedures is required to provide information to the patient about the necessity and benefits and possible side effects and alternative therapies of procedures. Then he gets the patient or his legal guardians, authorities or representatives to sign and fingerprint the informed consent and then make sure that the signature is present in the form, (In case the patient is alert and he can analyze things, the Informed consent could be obtained from him).

**Nurse**: The nurse is responsible for controlling the completion of the form for the patient before doing any procedures and make sure that the consent form is existent in each patient's profile.

Note: In case of emergency based on the physician’s opinion, if invasive medical procedure is seen as emergent with delay in it creating some harms for the patient, the doctor can treat the patient with a thorough description of the patient's situation and a mention of emergency reasons of doing medical procedure.

List of invasive procedures in the emergency ward which require the completion of informed consent:

### 2.3 Data Collection

In order to collect data, field work and participant observation were carried out for 515 hours from June 2014 to March 2016 in the emergency ward. Given (2008) states that: “Participant observation is a method of data collection in which the researcher takes part in everyday activities related to an area of social life in order to study an aspect of that life through the observation of events in their natural contexts [27].” According to Carspecken's recommendations, the researcher focused on the voice, vocal tone, posture, body movements, gestures, facial expression in her participant observations [28]. In the study, the question of participant observation was “how is the process of informed consent done in the emergency ward?” Then, based on the events and observed behaviors it was discussed with the people involved in order to clarify the underlying experiences, beliefs, values, and feelings. The duration of participant observations lasted 2-9 hours in different shifts and handwritten notes were taken by the researcher during observations. Conversations during observations were another source of data that were conducted personally or in groups. Also, face-to-face semi-structured interviews averaging 45 minutes were conducted. The time and place of interviews were selected by the participants themselves. Also, interviews were audio recorded with participants’ permission. In this way, the study experienced 14 semi-structured interviews with the patients and their attendants, 11 with physicians, 11 with nurses. Immediately after each section of field work, the researcher transcribed all of their notes.

The present study's interview questions were designed based on Carspecken’s protocol (1996). The protocol has four sections: Topic domain, lead-off question, covert category and probable follow up questions. Based on the protocol, the general range of interview topics is determined in the topic domain, then lead-off questions are designed to open up a topic domain. Carspecken believes that the best lead-off question is the concrete question about an event that the participant is some part of it and the researcher is observing it. Then, while the participant is describing the event, the researcher is trying to extract by asking suitable questions the background beliefs, values, and involved feelings on the event, these are termed as covert category. All in all, it's a good idea that the researcher has probable follow-up questions. Carspecken advises about the cases when interview time is very far from observation or in cases in which the researcher wants to just interview with the participant, it is better that the lead-off questions be about the events which happen routinely within the context or use of some descriptive (typical day) question such as “talk about your day at work” to be implemented [28].

According to the Carspecken's protocol, the interview questions of the present study are designed in Table **1**.

In order to meet trustworthiness of participant observation, thick description was used. It was done using a detailed description of observations and careful recording of time and context. The purpose was to describe the research field completely and explore the details of health care and patient's interactions and communications. Furthermore, facial expressions, participants' postures and movements were described in great detail. Denzin (1989), defines thick description as: “deep, dense, detailed accounts of problematic experiences... It presents details, context, emotions and the web of social relationships that join individuals to one another [29].” Thick description is a rich and wide collection of details about the methodology and context which make judgments possible about the fitness of research context in relation to other contexts [30, 31]. Moreover, prolonged engagement was considered as supportive methods for the trustworthiness of the study. According to the criteria introduced by Lincoln and Guba in 1994 as cited in Kawulich, if a researcher can prove that he has spent a considerable portion of time in the studied setting, his findings are considered to have an upper level of trustworthiness [32]. Furthermore, the researcher checked the interviews repeatedly and compared them with the observations. In this way, the agreement between results from recorded observations and interviews were reviewed and researcher's interpretations emerged. Also, in order to challenge her understanding, she used member check and peer check. Therefore, two experienced researchers verified results of the analysis, and a summary of the transcripts was shared with selected participants.

### 2.4 Ethical Considerations

The researcher, after receiving written permission from Ethics Committee of Tehran University of Medical Sciences, started to collect data. At first she was introduced to the educational hospital's supervisor through which was relayed to the head nurse of the emergency ward. The purpose of the study and data gathering strategies was described for the head nurse. Then the participation policy in the research field was explained and required coordination was done. At the same time, some probable questions and uncertainties of the participants were answered. Throughout the study a relationship based on trust and honesty was the aim of the researcher right from the initiation of the study. She assured them that secrecy as well as confidentiality of the information from data collection up to her publication would be kept. Also, in order to maintain ethical considerations, interviews were recorded after receiving consent from participants and the time and place of interviews were decided by the participants.

### 2.5 Data Analysis

Data analysis was done through conventional qualitative content analysis. In this approach, categories and themes were extracted directly from data. According to the framework of Elo and Kyngäs the main process includes three basic phases of preparation, organization and reporting. The purpose of the first phase is understanding and proximity to the content of the text. For this purpose, interviews, observations and documentations were read several times to gain a thorough perspective and a comprehensive overview. In the second phase of the analysis, decoding of the text was done. Then sub-categories of similar contents were grouped together and, finally, based on the researcher's idea, the main categories were abstracted from generic categories [25].

## 3 RESULTS

The characteristics of the participants are displayed in Tables **2** and **3**. In the process of analysis, 1329 codes emerged in three main categories: (1) A sense of frustration (2) Reverse protection, (3) Culture of paternalism in consent process (Table **4**). Generic categories and main categories have been presented below.

### 3.1 Main Category 1: A Sense of Frustration

Sense of frustration originated from a sense of being compelled, as well as learned helplessness. At the beginning of hospitalization, the patients or their attendants had to sign consent forms in order to allow healthcare providers to do medical treatment or surgical operation on them.

Also, they confirmed physician's acquittal of responsibility by their signature. In this way, sense of being compelled to sign the forms will ultimately lead to a sense of frustration and obedience in patients and their attendants. During field observations, when a patient’s attendant was asked about the reason for his unread signature of the form, he replied:

Patient’s attendant (A2): I have to sign it. If I do not sign it, they will probably not treat my patient and I have no other means except to sign it. I won't have a voice to get my rights. In fact, they do these things to stop objections so that nobody can object….. I will sign it unread because I have no choice.

The nurse in the emergency ward talks of his experiences of signing the form as a coercive act:

Nurse (N2): we had a patient whose attendant was a lawyer. The lawyer read the form and said: “I cannot sign and finger print the form by simply reading it (Surprised). I am not satisfied”. I told to him YOU HAVE TO SIGN IT. The lawyer finally signed and finger printed it, then he claimed that the way you receive consent forms, nobody reads them and everything given to the patient will be signed without reading.

In addition, the lack of accountability of healthcare providers leads to experiences of learned helplessness in the patients. Henceforth, health care providers do not answer many of the patients or their attendants’ questions; as a result, they prefer to sign without question every form that the medical staff provides. These situations happen frequently and lead to a sense of frustration in patients and their attendants. A nurse believed that:

Nurse (N5): If a patient asks too many questions, the physician replies to him /her “Why are you asking so many questions? (Meaning you speak more like a busybody). I told you once, if you do not want the treatment, get out” (Angrily).

A patient’s attendant talked of his experiences:

Patient’s attendant (A6): Every time I asked questions, they actually did not answer me, as a result, I stopped asking anymore questions about consent forms. I think all medical teams are irresponsible. They appear to be tired of people.

In summing up the main category of sense of frustration about consent, we can say that patients’ sense of being compelled along with experiences of learned helplessness leads to a sense of frustration in patients and their attendants.

### 3.2 Main Category 2: Reverse Protection

Reverse protection has been extracted from generic categories of patient disarmament and documentation. In the generic category of patient disarming, what is derived from data is that the reasons for designing consent forms and obtaining consent are to take the responsibility away from the physician and transfer the responsibility to the patient as well as depriving the patient of the right of complaining. It seems that with those strategies, the patient is disarmed, as a result, better protection is provided for the healthcare providers. The nurse believed the reasons for designing various consent forms were as follow:

Nurse (8): In fact consent forms provide protection for the physician and not the patient. Legally, it aims for the benefits of physicians and not the patients. They want to save themselves (physicians). Hence, as far as they can, they receive more forms signed and more fingerprints.

Furthermore, one of the strategies of protection provision for the medical team (because of their legal concerns) is documentation during the process of consent, which was done using different preventive techniques. One of the techniques involves frequent signatures received from patients and their attendants signed in the forms, notebooks and various medical documents. The following field note talks of the sample:

“It was 1:40 a.m. A young man came to the emergency ward complaining about chest pain. Upon checking his ECG (Electrocardiography) and lab tests, the emergency resident came to the conclusion that the patient had no heart problem and decided to discharge him. To do so, the emergency resident while speaking quickly told the patients’ attendants that “he has no problem and can be discharged”. However, the emergency resident asked them to sign some documents before leaving. To do so, the nurse in the patient's file asked for signatures of the patient, his wife and mother with the following words:

“I am (name of patient,) in spite of health team recommendations do not want to be hospitalized.”

It was also evident in the way the consent forms were received that they were designed to protect health care providers, the forms contained wordings such as *“the acquittal of healthcare team belonging”* which were not understandable to the patients but could provide legal guarantee for the medical team. The following field note is evidence for what was talked about:

I (researcher) brought the consent form to a patient’s attendant and asked him to read the form he had signed unread. The attendant read the form with a loud voice:

Patient’s attendant (A5): “we hereby announce the acquittal of the healthcare team belonging to this hospital”. What is this? I don't know what it is (reading the text again). Did I sign it? They told me to sign it and I did. I did it like a blind person (illiterate). Should I cancel it right now? (full of worries).

To sum up, the healthcare team on the process of consent generally tried to take the responsibility from the physician and transfer it to the patient using different methods. All of this was done to prevent the patient’s right to complain and disarm them. They, because of some legal worries, applied various strategies for documentation, so that they could provide more protection for themselves.

### 3.3 Main Category 3: Culture of Paternalism in Consent Process

This main category, which shows the dominant cultural concept in the emergency ward, was obtained from structural and organizational paternalism as well as medical paternalism.

Structural paternalism indicates that some of the characteristics related to the management structure of the field of study leads or entices health providers to provide paternalistic care in consent process, for example, lack of supervision on the process of informed consent, few personnel and crowded hospitals. However, the researcher's field observations indicate that even when there are few patients in hospitals and there are small loads of tasks for the healthcare team, again no explanations are given to the patient and their consent is not taken seriously.

Also, organizational culture in the emergency ward leads to a paternalistic trend in consent process by health care team. In this regard, the participants believed that the existing atmosphere in the ward is that of paternalistic. For example, when a patient’s attendant was asked if he was in a situation to be counseled about decisions related to the treatment or care given to his patient, he replied:

Patient’s attendant (P/A3): No, nothing. The behavior of the health care team is bossy, lordly and in an authoritarian way. It means we are servants, they are bosses.

The results also showed that paternalism in the consent process can relate to medical paternalism. The superior-inferior beliefs and behaviors of medical staff encompassed patient minimization, giving priority to physician's decision than to the patients’ preferences. Furthermore, the physicians’ use of an offensive and harsh manner in dealing with the patients is a behavior to exert power over them. This way, they force them to obey physician's prospective procedure using paternalistic tone. As an example, a supervisor in the emergency ward believed that:

Supervisor (N9): The patient cannot make any decisions in this emergency ward culture. The patient when coming to the ward must be obedient to every word we say, whatever we say, whatever we prescribe. The patient has no right to offer his ideas (emphatically) and generally they do not comment. Nobody should meddle with medical affairs. Whatever the doctor says, that is all and the patient should be obedient. The physician is the lone decision- maker.

A nurse, about the reasons of non-provision of information to the patient, believes:

Nurse (N6): our physicians think that if they speak more with the patients, their career status decreases. It is the physicians that should ask the patients questions (emphatically) and not the patient. A patient who asks more questions is very rude. Our physicians do not like to put themselves into trouble, the basis for the process is not well established yet. They feel despised when patient asks and they answer.

In this regard, a medical intern talked of his experiences:

Medical intern (Ph4): some patients, as an example, do not like to use Foley Catheter. If you ask them their preference, they will say no. However, if you start setting up the Foley, speaking sharply and behaving coldly and telling him that it is the physician's order and that your wants are of no importance, then he will give up and do nothing with any resistance and ultimately give in. When you act with more force, the outcome is even better than the time you act mercifully.

Another demonstration of paternalism seen frequently is the use of defective information upon consent. The use of technical terminology is an example of defective information. Patients’ attendants term the process as “encoded speech” to talk of conditions when healthcare providers use medical technical words.

In summing up the main category of culture of paternalism in consent process, we can say that structural and organizational culture in the unit empowers paternalism in it. In addition, the existing medical paternalism can play a role in this situation, with their superior views taking care of the patient and non-provision of useful and suitable information, all can endorse the existence of paternalism in obtaining consent.

Finally, it should be noted that in fieldwork, the researcher sometimes finds out that some healthcare members acted differently of what they did routinely in some cases. They supported patients and their role was to advocate for patients. For example, some of members facilitated patient's participation and provided thorough explanations about related procedures to the patients and let them decide on their own. The following field notes indicates this:

It is 18:00 and they are to do BMA (Bone Marrow Aspiration) to a patient. The physician is next to the patient, explaining about the diagnostic test and its probable side effects in full details. Then the doctor asks the unit secretary to bring consent form so that it is signed and sealed by the patient. In the following interview with the doctor he emphasized on the importance of paying due attention to the patient's rights:

Medical Resident (Ph9): the rights of the patient shouldn't be ignored. It's the duty of the nurse and physician to communicate with the patient. There is no excuse for not explaining the procedures to the patient, according to the laws and ethics in medicine. In the profession of medicine, if you are to apply on IV line (Intravenous Line) to a patient, you should get his consent and explain to him the reasons for its use, it is the patient's right. Any invasive procedures to be done on the patient, we should explain about. It take the least time. No one can claim that there is no time available. It takes no time to explain these words to the patient. It prevents of dangers, complaints and injuries, let it be for its ethical and conscience matters. As I told, one doctor should know about humanistic matters. He should first learn humanistic norms, them start doing medication.

Additionally, some of the health care members regarded sympathizing with the patient and his attendant and understanding their conditions as some necessary factor. In their justification, they referred to conscientious care and ethics. The nurse's attempt to provide cares based on humanistic norms is evident in the following interview:

Nurse (N1): a feeling based on the conscience tells that the patient and his attendance have their own rights. I tell to these newly employed nurses that the patient is expecting me to give him a hand and provide services to him. He is full of stress. It is better that we at least explain about the procedure we do for him. In fact, we should think the patient as a human being. One day, we ourselves will be on these hospital beds. Let's suppose the one lying on the bed is one of our dearest.

## 4 DISCUSSION

Informed consent is a process that provides the patient with ample information about possible outcomes, alternatives in treatment and its risks [33]. In this study, patients confront frustration. This condition is the result of a feeling of being compelled and learned helplessness; as a result, they do not attempt to ask more questions for clarification in the process of consent. The existence of feeling of being compelled and learned helplessness by patients in the current study can be an ethical hurdle in informed consent, since one of the principles of informed consent is lack of obligation (patients' understanding and their free agreement on treatment). Researcher's encounter with these participants' experiences while doing field work and her efforts to identify the concept, led her to the “learned helplessness”. This theory was introduced by Martin Seligman *et al* in 1965 [34]. It happens in conditions in which the person experiences some uncontrollable events and believes in the fact that he can do nothing to change the consequence and result of the events. This situation leads to a sense of indifference and decrease in motivation to start a new action [35]. Also, according to Berger (1983) as cited in UK essays, studies done on Seligman’s theory on students indicated that those who had suffered learned helplessness thought that all their efforts were futile, because in spite of their previous full efforts on a subject, they could never succeed, henceforth, they had decreased autonomy and felt that they had no control over their environment [34].

Reverse protection was the second main category in this study. It could be said that emphasis on informed consent processes is only a way to protect healthcare providers, undermines the role of them about patient advocacy. On the other hand, researchers believe that lack of concern towards patients' autonomy and change in the nature of forms to a medico-legal requirement [36, 37], degrades the implicit value of informed consent from an ethical practice for the patient to the legally protective action for the medical staff [38], so the result is an ample discrepancy between informed consent as a bio-ethical requirement and what is in reality perceived by patients [39]. O'Neill (2003), believes that institutions and professionals increasingly receive informed consent to protect themselves against, litigation, compensation claims, and accusation [40]. In the study by Procacciante and colleagues (2001) they concluded that informed consent has gradually turned into protector of physician's rights rather than guaranteeing patient rights, thus it has no real effect on patient's choice [36].

Culture of paternalism in consent process was the third main category resulting from structural and organizational paternalism as well as culture of medical paternalism. In this study, this characteristic is frequently seen in the form of superior- inferior beliefs and behaviors from medical staff to their patients during the consent procedure. Based on this belief, physicians chose to treat them toughly, coldly and aggressively in order to enforce their power onto the patients. Beauchamp and Childress (2001) wrote: “Paternalism is the intentional overriding of one person’s known preferences or actions by another person, where the person who overrides, justifies the action by the goal of benefiting or avoiding harm to the person whose preferences or actions are overridden” [1]. In paternalistic approach, it is believed that healthcare providers have special knowledge and professional elitism and this causes a power imbalance between them and patients in clinical situations, resulting in healthcare providers generally viewing themselves in a better and more superior position in relation to the patients [41, 42]]. In such perspectives, health care providers believe that they have an intuitive power and capability of interpreting the patient's condition and have gained this ability through frequent interactions with the patients [42], therefore, their professional knowledge will be sufficient as the lone decision- making tool for the benefit of the patient. According to this view, the patients and their attendants do not have the required literacy to make decisions and they are emotionally too nervous [33]. Meanwhile, it is believed in paternalism that the person cannot distinguish between his benefits and losses and is not able to maintain or promote them [41]. Hence, benefits of the patient are determined by healthcare providers, not by the patient receiving health services [43]. In fact, what paternalistic views ignore is the fact that they should pay to more than experience and scientific expertise when they are to decide on health of a person. Such decisions should consider factors such as patient's values, culture, beliefs, risk evaluation, economic considerations, the effect of decision on life style as well as the person's roles that could be unrelated to the physical integrity but affect the totality and wholeness of the person [33]. In other studies, there is also a mention of the paternalistic behaviors during consent process. As an example, in a study done by Lee and his colleagues (2009), the majority of participants had the feeling that physician-patient relationship was imbalanced and authoritarian beliefs of physicians had limited the patient asking questions during the rounds which leads to non-arbitrary decision- making by the patient [44]. In a study done by Abolfotouh and Adlan (2012), half the patients felt that their decisions are unimportant because it is the physician who decides for them. In the study, the majority of patients agreed to leave their decision making right to the physician. They said that saying no to the physician would mean disrupting their good relationship with the physician. The researchers interpreted the reason for such kinds of consent as lack of patients' awareness of their rights and the spread of medical paternalism in Saudi Arabia [38].

Furthermore, in present study, the information about the disease and treatments was mostly given to the patient as defective and conditional as possible. In other words, the consent of patients was not “informed”. Also, in this study, use of unknown and unfamiliar terminology in the consent forms and application of technical jargon by healthcare providers during the consent process can be attributed to the paternalistic care. Researchers claim even if healthcare providers' explanations are not clear for the patient, paternalism has happened [45]. Also, according to Beauchamp and Childress (2001): “Paternalistic acts typically involve force or coercion on the one hand, or deception, lying, manipulation of information, or nondisclosure of information on the other” [1]. The researchers at the disproportion of informed consent with medical paternalism, believe that they are in full contradiction because the primary aim of informed consent is to protect the patient welfare and promote his autonomy. Then as a result, there will be no harmony between autonomy, empowerment and awareness of patient in decision-making from one hand, and powerful paternalistic authority of medical staff on the other hand [46, 47]. In other studies, patients also could not comprehend the content of forms for the same reasons of using medical jargon and terms when explaining the problems to the patients [15-17, 48-50].

## 5 LIMITATIONS

The first author was responsible for collecting data in the study. Her experience in nursing may be considered as both the strengths and weaknesses of the study. Being able to understand medical jargons and talk to medical staff, enabled her to enter in medical team, and be accepted by them, but it may produce some biases. To raise awareness of researcher’s own biases, she reflected her preconceptions using peer check with the coauthors and supervisor. Also according to recommendations by Carspecken (1996), before entering the field and beginning data collection process she had a supervisor interview her on the things that she was expecting to find. Also discovering her biases continued by writing journal notes during field work [28].

We recognize that our data are collected from one hospital, which could limit generalizability to other hospitals, but the findings of this qualitative research may be transferable to other hospitals. Because transferability has been facilitated by researcher through thick description [51]. Hence, in the study, researcher attempted to increase the quality of thick description. It was done to pave the way for transferability of findings to other similar contexts and make findings more useful and meaningful.

## CONCLUSION

In summary the study illustrated that receiving informed consent in this emergency ward did not conform to important legal and ethical principles. The patients and their attendants had a feeling of being compelled and helplessness in obtaining consent process. Healthcare providers, due to their legal concerns, abused the consent form as an exculpation tool for protecting themselves. Structural and organizational paternalism established some difficulties in patient-centered care in the process of obtaining informed consent. Culture of medical paternalism with superior - inferior beliefs to the patient, did not basically consider any necessity for the patient's participation in decision-making process.

Considering the outcomes of this research, it is suggested that consent forms be rewritten and designed according to the patient's educational level and sociocultural context. In order to improve the consent process the supervision systems should be promoted. Furthermore, structural and organizational problems that facilitate paternalism should be reformed. Legal concerns of health care providers should also be identified, resolved or amended. In addition, medical staff should be trained on how to communicate with patients during consent process. In this way, the approaches of taking care of the patients in consent process will be changed from paternalistic approach to a patient-centered care while protecting patient’s autonomy.

The final point is the fact that the existence of legal and ethical challenges in consent process in the current research does not mean the ignorance of the whole effective service of health care providers. The aim is the presentation of an effective criticism, an attempt to correct the unwilling processes and provide a fundamental base for presenting more sufficient and desirable patterns so as to take better care of the patients.

## Figures and Tables

**Table:** 

***Invasive procedures***		***Invasive procedures***	
*1*	*chest tube insertion*	*8*	*Colonoscopy*
*2*	*CT scan and MRI*	*9*	*Bronchoscopy*
*3*	*Paracentesis*	*10*	*Endoscopic retrograde cholangiopancreatography*
*4*	*Thoracentesis*	*11*	*lumbar puncture*
*5*	*Tracheostomy*	*12*	*Central venous line insertion*
*6*	*Anesthesia*	*13*	*Blood transfusion*
*7*	*Pericardiocentesis*		

**Table 1:** Interview protocol.

**Interview protocol**			
**Topic domain**	**Lead-off questions**	**Covert category**	**Follow-up questions**
Strategies for the informed consent and its challenges	Today, I observed that …please explain what happened? Please picture us from hospitalization time up to now, what happened and how did they happen? (patient’s questions) Please talk about your typical day at work (physician or nurse’s question)	**Belief**: what do you think in this regard? How come you think in this way? **Acceptance**: Do you accept or not? Why? **Meaning**: what does this behavior mean to you? **Feeling**: what was your feeling? **Values**: why it should be this way? **Expectations**: what are your expectations, how should they be and How they shouldn't be.	Can you provide an example? What do you mean please elaborate more? What else do you remember in this regard?

**Table 2:** Characteristics of patients and their attendance.

**Code**	**Role**	**Gender**	**Age**	**Cause of hospitalization**	**Length of stay in ED (Hours)**
P1	Patient	Male	42	Acute kidney disease	5
P2	Patient	Male	52	Chest pain	1
P3	Patient	Male	62	Anemia	5
P4	Patient	Female	33	Appendicitis	3
A1	Patient’s attendant	Female	40	Fever	5
A2	Patient’s attendant	Male	45	Cholecystitis	2
A3	Patient’s attendant	Female	30	GI bleeding	7
A4	Patient’s attendant	Female	24	Chest pain	2
A5	Patient’s attendant	Male	43	Fever	8
A6	Patient’s attendant	Female	53	Decreased level of consciousness	48
P-A1	Patient- Attendant*	Male- Female	42-38	Severe Headache	5
P-A2	Patient- Attendant*	Female- Female	27-22	Appendicitis	3
P-A3	Patient- Attendant*	Male- Female	65-38	Cholecystitis	6
P-A4	Patient- Attendant*	Male-Male	28-43	Abdominal pain	4

P: Patient A: Patient’s attendant * In some cases, the conversations were conducted simultaneously with two or three participants.

**Table 3:** Characteristics of health care providers.

**Code**	**Role**	**Gender**	**Age**	**Job experience in hospital (Year)**
Ph1	Medical Intern	Female-Female-Female*	22-23-22	2-1.5-2
Ph2	Medical Intern	Male-Male-Male*	23-25-23	1.5-1.5-1.5
Ph3	Medical Intern	Female-Female*	23-24	1.5-1.5
Ph4	Medical Intern	Male	23	1
Ph5	Medical Intern	Female	22	1.5
Ph6	Medical Intern	Female	24	2
Ph7	Medical Resident	Female	29	5
Ph8	Medical Resident	Male	42	7
Ph9	Medical Resident	Male	46	12
Ph10	Emergency Specialist	Male	48	15
Ph11	Emergency Specialist	Male	49	14
N1	Nurse/ Nurse	Female-Female*	26-27	2-3
N2	Nurse	Female	29	7
N3	Nurse	Male	48	27
N4	Nurse/ Nurse	Male-Female*	38-35	17-9
N5	Nurse	Female	29	5
N6	Nurse/ Nurse	Male-Female*	38-36	17-8
N7	Nurse/ Nurse	Female-Female*	28-26	5-3
N8	Nurse	Male	48	28
N9	Nurse as supervisor	Male	50	30
N10	Nurse as supervisor	Female	49	25
N11	Head nurse	Male	52	28

Ph: Physician N: Nurse * In some cases, the conversations were conducted simultaneously with two or three participants.

**Table 4:** Generic categories and Main categories of the results.

**Generic Categories**	**Main Categories**
Sense of being compelled Learned helplessness	A sense of frustration
Patient disarmament Documentation Structural paternalism	Reverse protection
Organizational paternalism Medical paternalism	Culture of paternalism
